# PD21 Evidence Assessment Frameworks And Economic Modeling For Rare Diseases: A Stakeholder Survey Exploring Current Practices In HTA

**DOI:** 10.1017/S0266462325101566

**Published:** 2025-12-29

**Authors:** Martina Garau, Alicia Granados, Farzana Malik, Diana Sinkevich, Lea Wiedmann, Dorota Zgodka

## Abstract

**Introduction:**

Rare diseases (RDs) present unique challenges in health technology assessment (HTA) due to small, heterogeneous populations and limited clinical data, which complicate economic modeling and delay access to innovative interventions. This survey, conducted by the HTAi Rare Diseases Interest Group, explored key challenges in economic modeling for RDs, the application of solutions, and the use of evidence assessment frameworks in HTA practice.

**Methods:**

A mixed-method survey was developed with multiple-choice, Likert-scale, and open-text questions using the SurveyMonkey platform. Responses were collected from members of HTAi and the International Network of Agencies for Health Technology Assessment at conference sessions. Additional respondents were recruited via professional networks and direct emails. Options exceeding 50 percent of responses and rate weighted averages guided the interpretation and identification of areas of agreement.

**Results:**

In total, 36 individuals across all continents, mostly from consultancy and HTA agencies, responded to the survey. Key challenges in economic modeling included insufficient quality of clinical trials (duration and sample size), scarcity of robust data, and lack of health-related quality of life metrics. The most common solutions included using proxy diseases, pooled data, and qualitative research. Broader impacts were integrated through societal perspectives, decision modifiers, and deliberative processes. GRADE was the most commonly cited explicit evidence assessment framework. In some cases, implicit approaches dominated HTA practice where challenges such as single-arm trials and reliance on real-world evidence prevailed, though patient-reported outcomes had gained some acceptance.

**Conclusions:**

Scarcity and quality of clinical data remain significant barriers to economic modeling for RDs. Solutions such as qualitative methods and pooling data show promise. The broader impacts of RDs are increasingly being considered, but further research is essential to refine methods, embrace non-traditional data, and develop appropriate frameworks to assess evidence for RDs in HTA.

